# A radiomics-based interpretable machine learning model to predict the HER2 status in bladder cancer: a multicenter study

**DOI:** 10.1186/s13244-024-01840-3

**Published:** 2024-10-28

**Authors:** Zongjie Wei, Xuesong Bai, Yingjie Xv, Shao-Hao Chen, Siwen Yin, Yang Li, Fajin Lv, Mingzhao Xiao, Yongpeng Xie

**Affiliations:** 1 Department of Urology, The First Affiliated Hospital of Chongqing Medical University, Chongqing, China; 2 Department of Urology, Urology Research Institute, The First Affiliated Hospital of Fujian Medical University, Fuzhou, China; 3 Department of Urology, National Region Medical Center, Binhai Campus of the First Affiliated Hospital, Fujian Medical University, Fuzhou, China; 4 Department of Urology, Chongqing University Fuling Hospital, Chongqing, China; 5 Department of Urology, Chongqing University Three Gorges Hospital, Chongqing, China; 6 Department of Radiology, The First Affiliated Hospital of Chongqing Medical University, Chongqing, China

**Keywords:** Radiomics, Bladder cancer, Computed tomography, HER2, Machine learning

## Abstract

**Objective:**

To develop a computed tomography (CT) radiomics-based interpretable machine learning (ML) model to preoperatively predict human epidermal growth factor receptor 2 (HER2) status in bladder cancer (BCa) with multicenter validation.

**Methods:**

In this retrospective study, 207 patients with pathologically confirmed BCa were enrolled and divided into the training set (*n* = 154) and test set (*n* = 53). Least absolute shrinkage and selection operator (LASSO) regression was used to identify the most discriminative features in the training set. Five radiomics-based ML models, namely logistic regression (LR), support vector machine (SVM), k-nearest neighbors (KNN), eXtreme Gradient Boosting (XGBoost) and random forest (RF), were developed. The predictive performance of established ML models was evaluated by the area under the receiver operating characteristic curve (AUC). The Shapley additive explanation (SHAP) was used to analyze the interpretability of ML models.

**Results:**

A total of 1218 radiomics features were extracted from the nephrographic phase CT images, and 11 features were filtered for constructing ML models. In the test set, the AUCs of LR, SVM, KNN, XGBoost, and RF were 0.803, 0.709, 0.679, 0.794, and 0.815, with corresponding accuracies of 71.7%, 69.8%, 60.4%, 75.5%, and 75.5%, respectively. RF was identified as the optimal classifier. SHAP analysis showed that texture features (gray level size zone matrix and gray level co-occurrence matrix) were significant predictors of HER2 status.

**Conclusions:**

The radiomics-based interpretable ML model provides a noninvasive tool to predict the HER2 status of BCa with satisfactory discriminatory performance.

**Critical relevance statement:**

An interpretable radiomics-based machine learning model can preoperatively predict HER2 status in bladder cancer, potentially aiding in the clinical decision-making process.

**Key Points:**

- The CT radiomics model could identify HER2 status in bladder cancer.
- The random forest model showed a more robust and accurate performance.
- The model demonstrated favorable interpretability through SHAP method.

**Graphical Abstract:**

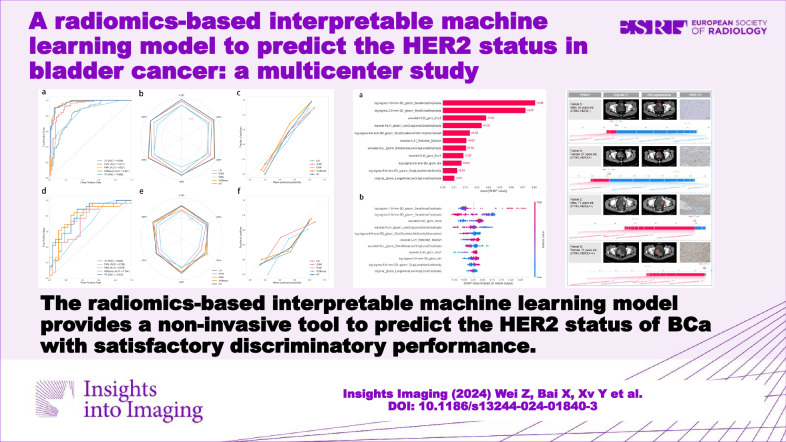

## Introduction

Bladder cancer (BCa) is a common malignant tumor of the urinary system, characterized by easy recurrence, rapid progression, and metastasis [1, 2]. Despite undergoing transurethral resection of bladder tumor and intravesical chemotherapy or Bacillus Calmette-Guérin (BCG) therapy, more than 50% of non-muscle invasive bladder cancers still experience recurrence and may eventually progress to muscle-invasive bladder cancer (MIBC) [3]. Patients with MIBC have a poor prognosis, with a 5-year overall survival rate of less than 50% [4].

In recent years, the rapid development of novel targeted treatments has provided hope for precise personalized treatment of BCa [5]. BCa stands as the third most prevalent cancer exhibiting human epidermal growth factor receptor 2 (HER2) overexpression, making it a potential therapeutic target [6]. Meanwhile, HER2 plays a key role in driving tumorigenesis, and high expression is often associated with pathological malignancy and poor prognosis in BCa [7, 8]. Ongoing phase II and phase III clinical studies have shown encouraging promise for the efficacy and safety of ADC drugs targeting HER2 in the treatment of BCa [9–11]. An open-label, phase II, multicenter clinical trial involving 107 patients showed that disitamab vedotin, a novel ADC drug targeting HER2, exhibited good efficacy (with an objective response rate of 50.5%) and manageable safety profiles in patients with HER2-positive locally advanced or metastatic urothelial carcinoma who had received at least one line of systemic chemotherapy [9]. Recently, the FDA has approved disitamab vedotin for the treatment of advanced urothelial carcinoma [12]. Therefore, accurate discrimination between HER2-positive (HER2-p) and HER2-negative (HER2-n) in patients with BCa is essential for individualized treatment strategies.

The current primary method for evaluating HER2 expression status is immunohistochemistry (IHC) staining of transurethral cystoscopy biopsy or surgical specimens [13]. However, due to intra-tumor heterogeneity and sampling limitations, partial specimens may not comprehensively represent the entire tumor. Moreover, this traditional method is invasive, time-consuming, and lacks reproducibility. Therefore, there is an urgent need for a noninvasive and comprehensive method to accurately assess the HER2 status in BCa.

Radiomics is a novel high-throughput quantitative image analysis method that can rapidly and accurately extract and analyze information from CT, MRI, and other medical images, providing a noninvasive and repeatable tool for identifying entire tumor heterogeneity [14, 15]. According to previous studies, radiomics can accurately assess the biological behavior of bladder tumors, including the identification of benign and malignant tumors, tumor grading, tumor staging and tumor recurrence [16–18]. Meanwhile, radiomics has also been used to accurately and noninvasively predict the expression status of HER2, PD-1, and Ki-67 in breast, gastric, and liver cancers using preoperative imaging data [19–24]. To the best of our knowledge, it is the first multicenter study based on CT scans to explore the potential of radiomics in predicting HER2 expression status in BCa.

Therefore, the aims of this multicenter study were to develop and validate a machine learning (ML) model based on enhanced CT radiomics features for predicting HER2 status in BCa. In addition, the study aimed to explore the interpretability of the ML radiomics model by the application of Shapley additive explanation (SHAP).

## Materials and methods

### Patients

**Fig. 1:**
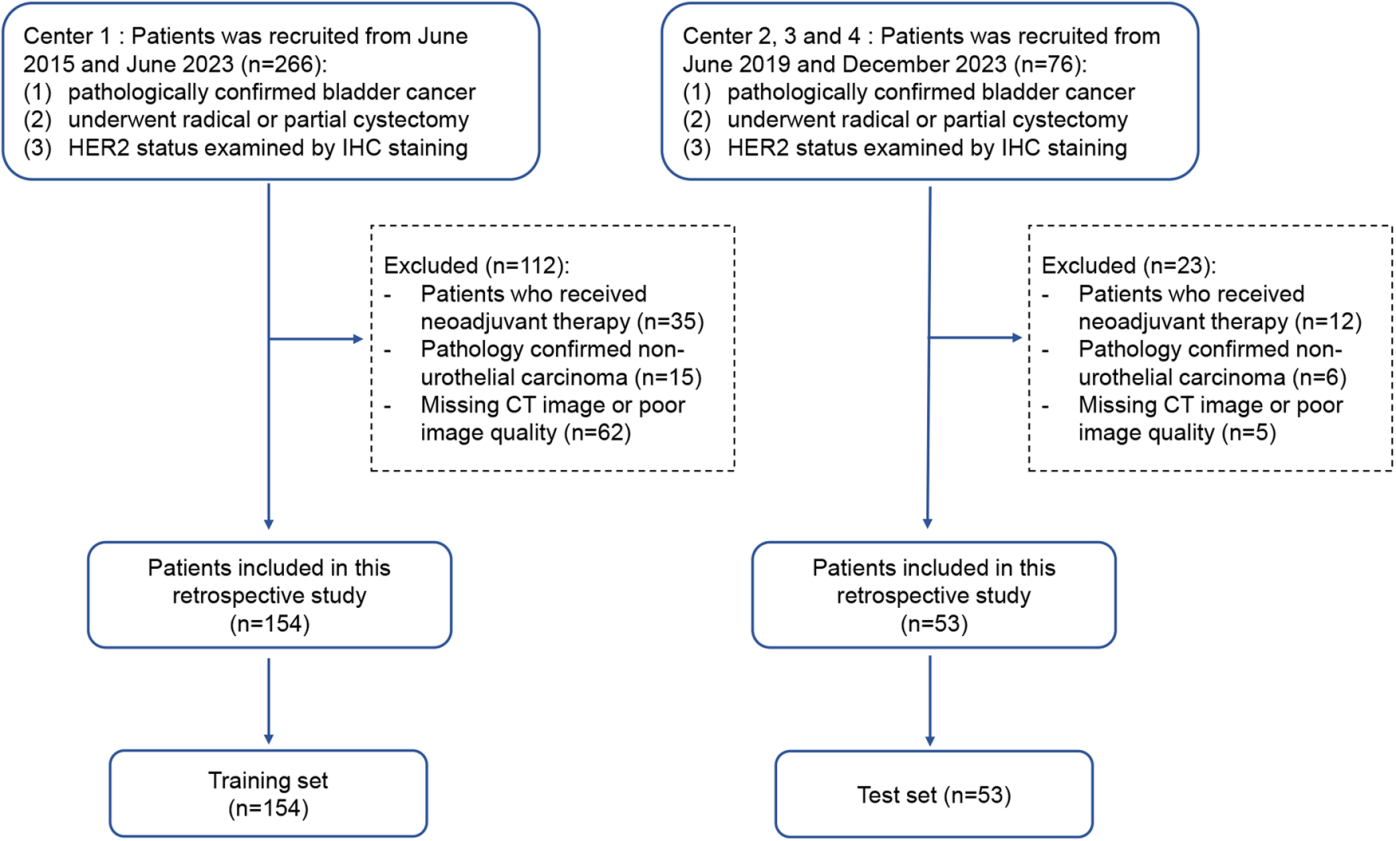
The flowchart of the patient recruitment process

The patients who underwent radical cystectomy or partial cystectomy with pathologically confirmed BCa were retrospectively recruited from four independent hospitals. The inclusion criteria were: (1) patients with pathologically confirmed BCa; (2) patients who underwent radical cystectomy or partial cystectomy; (3) contrast-CT scans available within 2 weeks before surgery. The exclusion criteria were (1) received neoadjuvant therapy; (2) pathologically confirmed non-urothelial carcinoma; (3) incomplete CT data or poor image quality. Finally, a total of 154 patients were enrolled from the primary center between June 2015 and June 2023 as the training set for model construction. In the test set, we retrospectively enrolled 53 patients with BCa between June 2019 and December 2023 from the other three centers. The flowchart of the patient recruitment process is shown in Fig. 1. This study was approved by the institutional review board of our institutions, and the requirement for informed consent was waived. This study has been reported in line with the transparent reporting of a multivariable prediction model for individual prognosis or diagnosis (TRIPOD) statement and in accordance with the Declaration of Helsinki [25].

The following clinical and pathological data were collected from the electronic medical records: age, gender, pathological T-stage, pathological N-stage, and pathological grade. Pathologic tumor staging was based on the eighth edition of the American Joint Committee on Cancer Staging System [26].

### Assessment of HER2 status

HER2 status was assessed by IHC assay performed on formalin-fixed, paraffin-embedded surgical specimens. The samples were scored based on the 2018 American Society of Clinical Oncology HER2 testing guideline by two pathologists who were blinded to the clinical data [27]. IHC staining results were scored as follows: 0, no staining or less than 10% of tumor cells stained; 1, more than 10% of cells exhibited weak and partial membrane staining; 2, more than 10% of cells showed weak to moderate, complete membrane staining; 3, more than 10% of tumor cells displayed strong, complete membrane staining. HER2-p was defined by IHC scores of 2+ or 3+, while HER2-n was defined by scores of 0 or 1+.

### Image collection and ROI segmentation

All patients underwent enhanced CT scan within 2 weeks before surgery. The details of the CT scanners and scanning parameters for the different institutions are shown in Table S1. The CT scan images were downloaded from Picture Archiving and Communication Systems (PACS) and saved in the original Digital Imaging and Communications in Medicine (DICOM) format. In this study, the nephrographic phase (NP) CT image, the most common imaging for tumor identification in bladder cancer, was chosen for subsequent analysis.

A urological radiologist with 5 years of experience (Reader A), who was blinded to pathological clinical information, manually segmented the region of interest (ROI) of the tumors via ITK-SNAP software (version 3.6.0, http://www.itksnap.org). For multiple tumors, the lesion with the largest diameter was chosen for ROI segmentation and subsequent feature extraction. Thirty images were randomly selected for ROI segmentation by Reader A after 2 weeks of the first segmentation and Reader B (with more than 10 years of experience in the diagnosis of genitourinary diseases), respectively, in order to evaluate inter- and intra-observer reproducibility of the radiomics feature extraction. Radiomics features with inter- and intra-class correlation coefficient (ICC) > 0.75 were considered to exhibit strong reliability and were consequently retained for model construction.

### Radiomics feature extraction and selection

Radiomics features were extracted from the 3D ROIs of each patient’s CT images using the open-source pyradiomics package (version 2.2.0) in Python. The details of the radiomics feature extraction are shown in Supplementary Material: Appendix E1. All radiomics features were standardized separately using z-scores normalization. Several feature selection methods were used to further reduce overfitting and improve the robustness of the model. Feature selection was performed in the training set according to the following steps: (1) the stable features with ICC > 0.75 were selected for further analysis; (2) using Spearman correlation analysis to filter out redundant features. (3) Features with significant differences in the HER2-p and HER2-n groups were selected using independent samples *t*-test; (4) the least absolute shrinkage and selection operator (LASSO) regression algorithm with fivefold cross-validation was used to further eliminate irrelevant features.

### Model construction

**Fig. 2:**
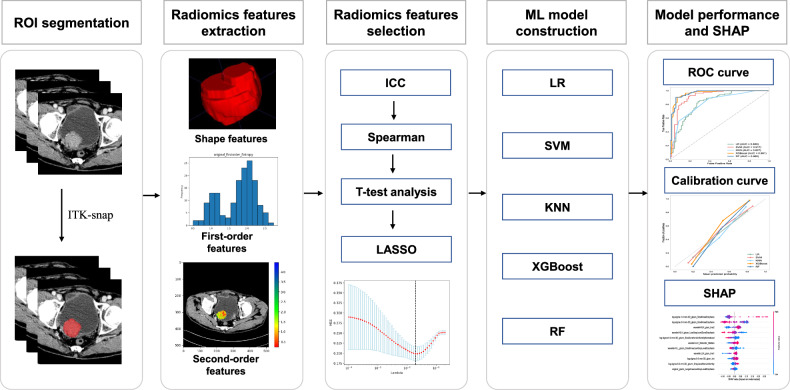
The overall workflow of this study. CT, computed tomography; ICC, inter- and intra-class correlation coefficient; ROI, region of interest; ROC, receiver operating characteristic; SHAP, Shapley additive explanation; LASSO, the least absolute shrinkage and selection operator

Five commonly used ML algorithms were used to construct predictive models in the training set, including logistic regression (LR), random forest (RF), support vector machine (SVM), extreme gradient boosting (XGBoost), and k-nearest neighbors (KNN). The receiver operating characteristic (ROC) curve and the area under the ROC curve (AUC) value were used to evaluate the discrimination performance of established ML models. Delong’s test was used to compare the AUC between models. The cutoff value identified by Youden index was used to calculate the accuracy, sensitivity, specificity, positive predictive value (PPV), and negative predictive value (NPV). Model construction and performance evaluation were performed in Python 3.8.0. The overall workflow of this study is summarized in Fig. 2.

### Model interpretation

The SHAP, as a model interpretation method based on game theory, can provide insights into the influence of each feature on model predictions by calculating the contribution of each feature [28]. It can obtain a global interpretation and a local interpretation for each sample. In this study, we used the SHAP method to interpret the constructed ML model, addressing the “black box” challenge. All analyses were conducted using the SHAP package (version 2.0.0) in Python. SHAP feature importance plots and summary plots were generated. A few representative cases were selected to create SHAP force plots to better understand the model’s predictions.

### Statistical analysis

All statistical analyses were performed using the R package (version 4.1.2; https://www.r-project.org) and SPSS statistical software. Continuous variables were presented as the means and standard deviation (SD), and categorical variables were presented as frequencies and percentages; 95% confidence intervals (CI) were calculated using the bootstrapping method. Clinical characteristics between training and test sets were compared by using the chi-square test (or Fisher exact test) and *t*-tests (or Mann–Whitney *U* test), respectively. All analyses were considered statistically significant with a two-sided *p*-value < 0.05.

## Results

### Clinicopathological data

Patient characteristics are summarized in Table 1. A total of 207 BCa patients were included in the study, with 109 (52.7%) having HER2-p status and the remaining 98 (47.3%) having HER2-n status. No significant differences were observed between the two cohorts in terms of age, gender, or pathological grade. However, there were significant differences between the training and test sets in terms of pathological T-stage (*p* = 0.003) and pathological N-stage (*p* = 0.024).

**Table 1:** Comparison of clinical and pathological characteristics of the training and test sets

Characteristic	Total (*n* = 207)	Training set (*n* = 154)	Test set (*n* = 53)	*p*-value
Age	67.52 ± 10.32	67.41 ± 9.80	67.85 ± 11.80	0.790
Gender				< 0.850
Male	185 (89.4%)	138 (89.6%)	47 (88.7%)	
Female	22 (10.6%)	16 (10.4%)	6 (11.3%)	
Pathological T-stage				0.003
Ta	10 (4.8%)	10 (6.5%)	0	
T1	58 (28.0%)	52 (33.8%)	6 (11.3%)	
T2	79 (38.2%)	50 (32.5%)	29 (54.7%)	
T3	39 (18.8%)	27 (17.5%)	12 (22.6%)	
T4	21 (10.1%)	15 (9.7%)	6 (11.3%)	
Pathological N-stage				0.024
N0/Nx	194 (93.7)	148 (96.1%)	46 (86.8%)	
N1-2	13 (6.3%)	6 (3.9%)	7 (13.2%)	
Pathological grade				0.093
Low	20 (9.7%)	18 (11.7%)	2 (3.8%)	
High	187 (90.3%)	136 (88.3%)	51 (96.2%)	
HER2 status				0.543
Positive	109 (52.7%)	83 (53.9%)	26 (49.1%)	
Negative	98 (47.3%)	71 (46.1%)	27 (49.1%)	

### Feature selection

A total of 1218 radiomics features were extracted from nephrographic phase CT for each patient; 887 radiomics features showed good inter- and intra-observer agreement with ICC > 0.75, and were used for further analysis; 223 radiomics features were retained after removing highly correlated features and 33 radiomics features were retained after *t*-test analysis. Finally, the LASSO logistic regression method with fivefold cross-validation was performed, and eleven features were screened to establish the radiomics model (Fig. S1). Detailed descriptions of the selected radiomics features are provided in Supplementary Material: Appendix E2. The heatmap and correlation heatmap of these features are shown in Figs. S2 and S3.

### Model performance

Selected features were fed into five ML models. The hyperparameters for each model used in the study are shown in Supplementary Material: Appendix E3. The performance of ML models in the training and test sets is shown in Table 2. In the training set, the AUC values were 0.829 (95% CI: 0.759–0.893) for LR, 0.917 (95% CI: 0.863–0.961) for SVM, 0.827 (95% CI: 0.761–0.883) for KNN, 0.961 (95% CI: 0.927–0.987) for XGBoost and 0.965 (95% CI: 0.933–0.987) for RF. The results of Delong’s test showed that the AUC of RF exhibited significant differences compared to LR (*p* < 0.001), SVM (*p* = 0.035), and KNN (*p* < 0.001), whereas no significant difference was observed between RF and XGBoost (*p* = 0.643) (Table S2). Although there was no significant difference in AUC, RF achieved higher accuracy, sensitivity, PPV, and NPV compared to XGBoost.

**Table 2:** Comparison of the performance of ML models in training and test sets

Models		AUC	ACC	SEN	SPE	PPV	NPV
Training set	LR	0.829 (0.759–0.893)	0.779	0.718	0.831	0.775	0.785
	SVM	0.917 (0.863–0.961)	0.864	0.817	0.904	0.852	0.879
	KNN	0.827 (0.761–0.883)	0.734	0.775	0.699	0.784	0.688
	XGBoost	0.961 (0.927–0.987)	0.909	0.93	0.892	0.937	0.88
	RF	0.965 (0.933–0.987)	0.916	0.944	0.892	0.949	0.882
Test set	LR	0.803 (0.670–0.913)	0.717	0.741	0.692	0.72	0.714
	SVM	0.709 (0.554–0.847)	0.698	0.556	0.846	0.647	0.789
	KNN	0.679 (0.516–0.815)	0.604	0.481	0.731	0.576	0.65
	XGBoost	0.794 (0.656–0.911)	0.755	0.704	0.808	0.724	0.792
	RF	0.815 (0.695–0.917)	0.755	0.667	0.846	0.710	0.818

*AUC* area under the receiver operating characteristic curve, *ACC* accuracy, *SEN* sensitivity, *SPE* specificity, *PPV* positive predictive value, *NPV* negative predictive value

**Fig. 3:**
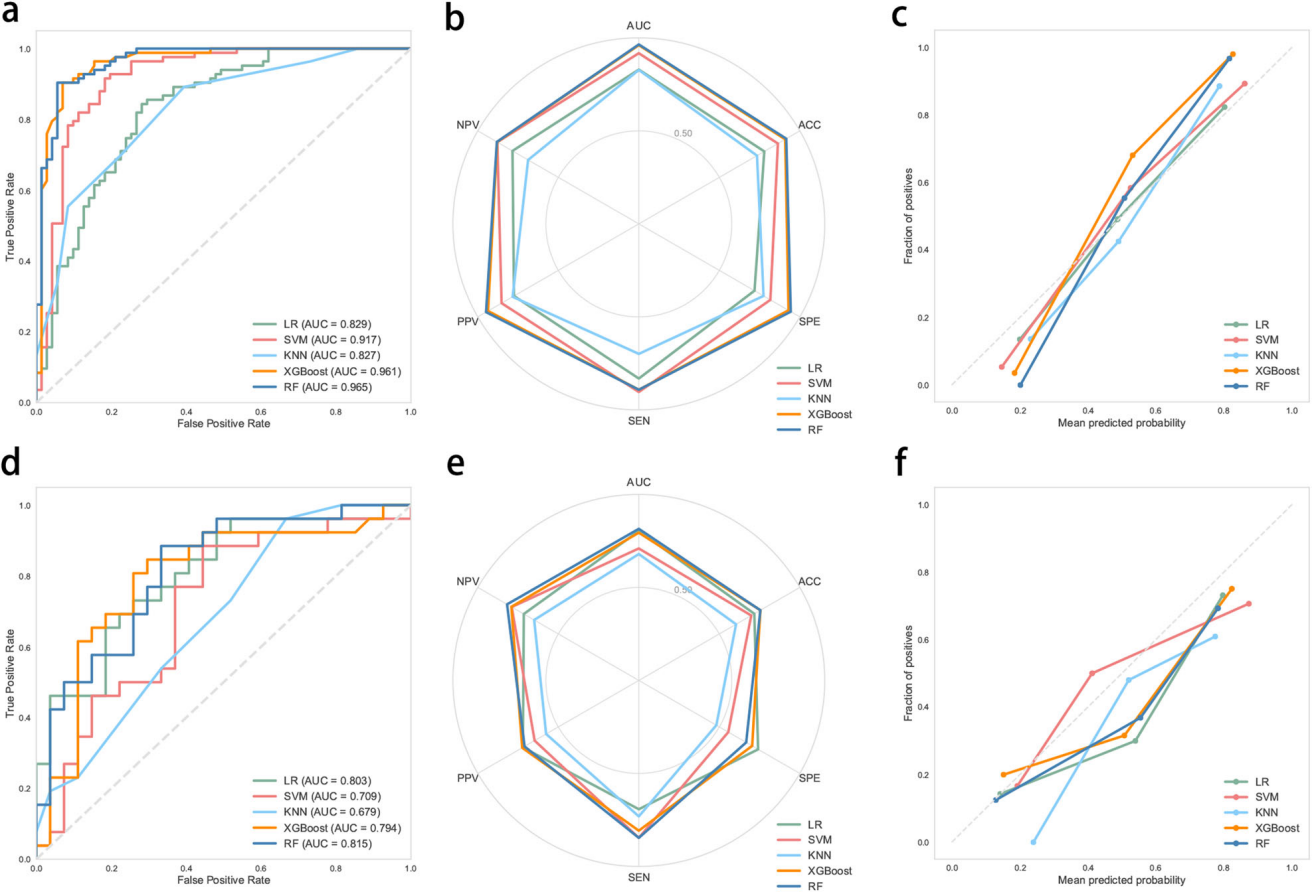
Performance comparison of ML models for HER2 prediction in bladder cancer patients. **a**–**c** Displays the ROC curve, performance radar chart and calibration curve of the ML models in the training set, respectively. **d**–**f** Displays the ROC curve, performance radar chart and calibration curve of the ML models in the test set, respectively. AUC, area under the curve; ML, machine learning; ROC, receiver operating characteristic

In the test set, the performance of RF (AUC of 0.815) outperformed LR (AUC of 0.803), SVM (AUC of 0.709), KNN (AUC of 0.679) and XGBoost (AUC of 0.794). The RF model had an accuracy of 0.755, sensitivity of 0.667, specificity of 0.846, PPV of 0.710, and NPV of 0.818 in the test set. Therefore, RF was chosen as the final model. In addition, we presented the ROC curves, performance radar plots and calibration curves for all models in the training and testing sets (Fig. 3).

### Model interpretability

**Fig. 4:**
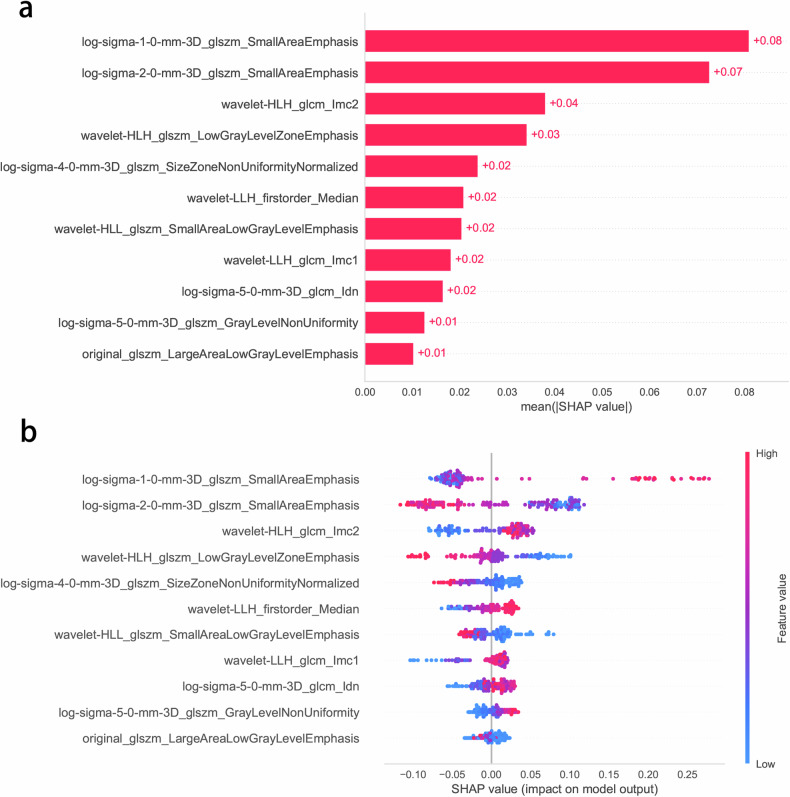
The interpretability of the ML radiomics model was assessed using the SHAP method. **a** The SHAP bar chart shows the importance of each feature based on the mean SHAP values. **b** The SHAP summary plot shows the impact of each feature on the model predictions. Individual dots symbolize patients, and different colors represent different levels of influence on the model output. ML, machine learning; SHAP, Shapley additive explanation

**Fig. 5:**
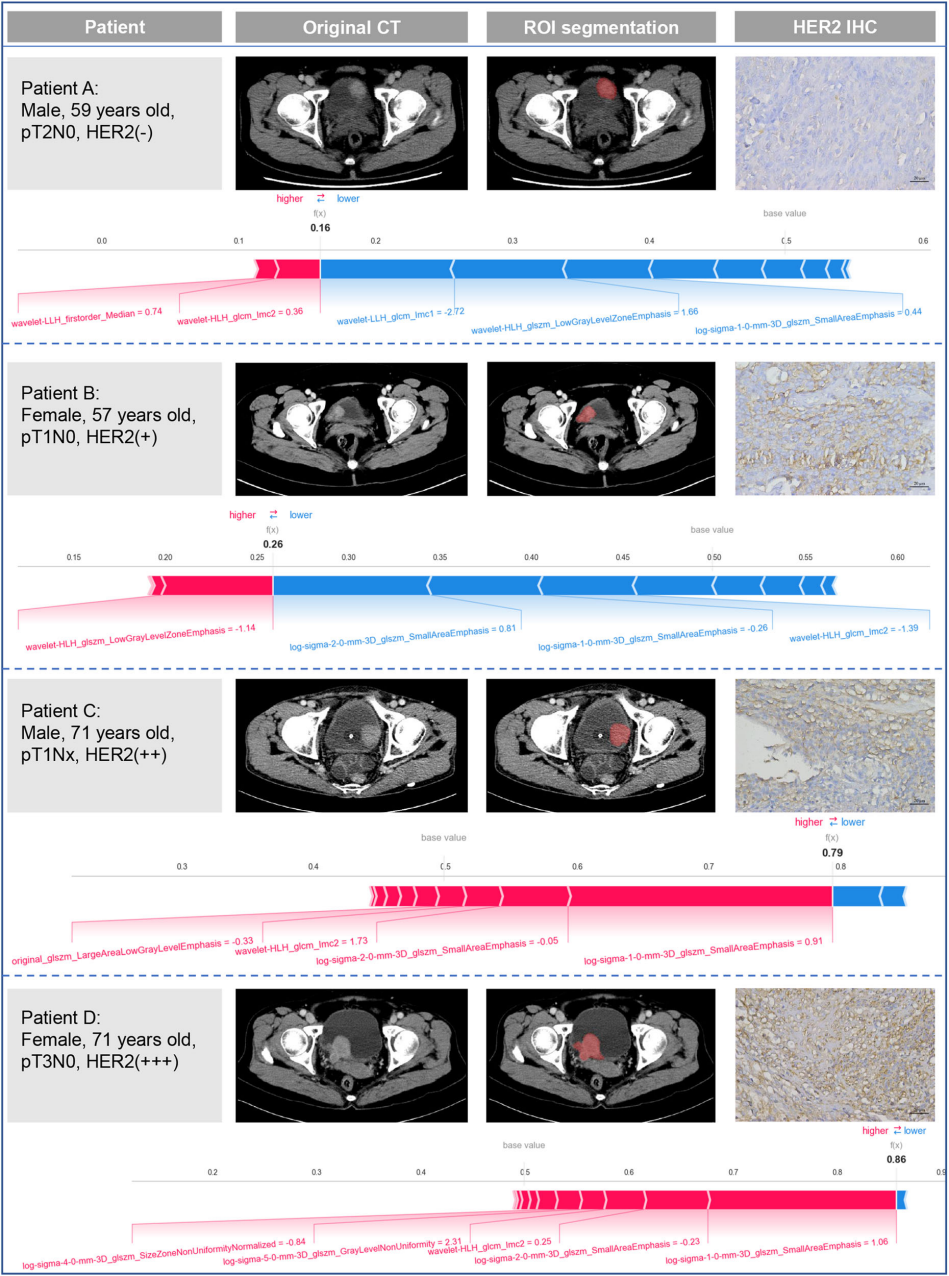
Four representative cases correctly predicted as HER2-negative (patients A and B) and HER2-positive (patients C and D) were individually visualized by SHAP method. SHAP, Shapley additive explanation

We calculated SHAP values for each radiomics feature in the RF model. In the global interpretation, the SHAP bar graph (Fig. 4a) showed the importance of each feature. The results showed that log-sigma-1-0-mm-3D_glszm_SmallAreaEmphasis and log-sigma-2-0-mm-3D_glszm_SmallAreaEmphasis contributed the most to the RF model. We depicted the summary plot of SHAP value (Fig. 4b), which explained the impact of each feature on the model predictions. Figure 5 shows four typical cases of correctly predicted HER2 positive and negative. To enhance the understanding of model decisions, two decision trees within the RF model are illustrated in Fig. S4.

## Discussion

In this study, we developed and externally validated an interpretable ML model using radiomics features extracted from enhanced CT scans to predict HER2 expression status in BCa. In recent years, HER2-targeted therapy has shown efficacy in BCa. However, the heterogeneity among pathological tissues poses challenges for the accurate interpretation of HER2 status using traditional IHC methods. Our radiomics-based model can extract features from the entire tumor region and has exhibited robust performance in differentiating HER2-positive from HER2-negative diseases. This model can noninvasively predict HER2 expression status preoperatively, potentially improving patient outcomes by enabling earlier and more precise intervention strategies. To our knowledge, this is the initial attempt to construct a noninvasive predictive model for HER2 expression in BCa using enhanced CT-based radiomics features and ML algorithms.

Recently, radiomics-based image analysis techniques are rapidly advancing, providing a noninvasive and automated approach for tumor diagnosis, staging and prognosis prediction. Yu et al developed a radiomics ML model based on MRI images to predict HER2 status in patients with urothelial bladder cancer [29]. The SVM achieved an AUC of 0.886 and 0.712 in the validation and test cohorts, respectively. Although MRI has better soft tissue resolution compared to CT, it is limited by its higher cost, longer scanning time, and incompatibility with certain metal implants or claustrophobic patients. Therefore, the potential application value of CT for predicting HER2 status in BCa is still worth exploring. In previous literature, CT-based radiomics models have also been constructed to predict PD-L1 and Ki-67 expression status in bladder cancer [22, 23]. In a previous study by Cao et al, ML models based on radiomics features extracted from CT scans achieved an AUC ranging from 0.753 to 0.766 in the validation set for predicting PD-L1 expression status in BCa [23]. Feng et al reported that CT radiomics nomogram combined clinical factors performed well in predicted Ki-67 expression in BCa, with an AUC of 0.887 in the validation set [22]. Their analyses uncovered a potential association between CT-based radiomics features (including shape, first order and texture features) and the pathobiological characteristics of BCa. However, none of these studies included patients from external centers to validate the model’s generalizability and stability. Our study also demonstrated that radiomics features extracted from enhanced CT images can be used to predict HER2 status in BCa. In this study, eleven optimal features (including first-order, gray level co-occurrence matrix (GLCM), and gray level size zone matrix (GLSZM) features), which were mainly associated with tumor heterogeneity, were ultimately selected for modeling. We evaluated five commonly used ML models and found that RF exhibited superior performance in an independent test set, with an AUC of 0.815. The performance of our model was comparable to previous studies using radiomics methods to predict HER2 expression in breast and gastric cancer [19–21, 30]. This study demonstrated that radiomics-based ML methods can noninvasively assess HER2 status in BCa, which is especially valuable for patients who cannot undergo invasive procedures.

The inherent “black-box” nature and limited interpretability of ML models prevent their widespread use in clinical practice, with clinicians often showing hesitation to trust their predictions [31]. First, through theglobal interpretability of the SHAP method, we identified log-sigma-1-0-3D_glszm_SmallAreaEmphasis, log-sigma-2-0-3D_glszm_SmallAreaEmphasis and wavelet-HLH_glcm_lmc2 as important predictors of HER2 status. GLSZM and GLCM are commonly used features for describing image texture and provide information about tumor heterogeneity by calculating the spatial relationships between pairs of pixels or voxels with different grayscale intensities. “Small Area Emphasis” measures the distribution of small-scale zones and can describe the fineness of the texture. A previous study reported that the “Small Area Emphasis” feature is an important predictor of HER2 expression in gastric cancer [32]. Additionally, in terms of local interpretability, we visualized the SHAP force plots for individual instances, showing how individual features influence the prediction process. Furthermore, we also visualized individual trees within the RF model, providing an intuitive understanding of the decision paths and logic in the prediction process. This approach helps clinicians understand the internal mechanisms and decision-making logic of ML models, which is crucial for gaining trust and acceptance in clinical settings.

This study also has some limitations. First, due to the retrospective nature of this study, our sample size was relatively small and inevitably subject to selection bias, despite the data collection from multiple centers. Furthermore, there were differences in some baseline characteristics (such as pathological T-stage and N-stage) between the training and test sets, which may affect the study outcome. Therefore, the findings of this study need further verification in large sample sizes and prospective cohorts. Second, although we interpreted the model predictions using the SHAP algorithm, further research is needed to investigate the underlying biological connections between radiomics and genomics. Finally, manual segmentation of ROIs by radiologists is a time-consuming process and may introduce subjectivity and variability. Therefore, future research should focus on advanced deep learning-based automatic segmentation and end-to-end techniques to minimize human intervention, ensuring reproducibility and efficiency.

## Conclusion

In conclusion, radiomics features based on contrast-enhanced CT offer a novel noninvasive approach to predict the HER2 status of BCa. A radiomics-based interpretable ML model exhibited satisfactory discrimination performance and may be helpful in the clinical decision-making process.

## Supplementary information

ELECTRONIC SUPPLEMENTARY MATERIAL

## Data Availability

The datasets analyzed during the current study are not publicly available due to the need for follow‑up research but are available from the corresponding author upon reasonable request.

## Abbreviations

AUC: Area under the receiver operating characteristic curve
BCa: Bladder cancer
GLCM: Gray level co-occurrence matrix
GLSZM: Gray level size zone matrix
HER2: Human epidermal growth factor receptor 2
ICC: Inter- and intra-class correlation coefficient
LASSO: Least absolute shrinkage and selection operator
MIBC: Muscle invasive bladder cancer
ML: Machine learning
NPV: Negative predictive value
PPV: Positive predictive value
ROC: Receiver operating characteristic
ROI: Region of interest
SHAP: Shapley additive explanation
