# Supplementary material for: A radiomics-based interpretable machine learning model to predict the HER2 status in bladder cancer: a multicenter study
Source: Insights Imaging. 2024 Oct 28;15:262. doi: 10.1186/s13244-024-01840-3 (PMC11519251; doi:10.1186/s13244-024-01840-3)
Supplement: Supplementary file 1 — ELECTRONIC SUPPLEMENTARY MATERIAL [file 13244_2024_1840_MOESM1_ESM.pdf]

A radiomics-based interpretable machine learning model to predict the HER2 status in bladder cancer: a multicenter study

ELECTRONIC SUPPLEMENTARY MATERIAL

Appendix E1: The details of radiomics feature extraction of this study

Before radiomics features extraction, all images were resampled to symmetrical voxels of 1*1*1 mm3 using spline interference. CT-based feature extraction was performed using the pyradiomics package (version 2.2.0) in Python (version 3.7.1), according to the guidelines of the Image Biomarker Standardization Initiative (IBSI).

Six kinds of radiomics features were extracted in this study:

(1) First-order statistics: describes the distribution of voxel intensities within ROIs by common and basic metrics.

(2) Shape-based features: describe the size and shape of ROIs.

(3) Grey Level Co-occurrence Matrix (GLCM): describes the spatial relationship between pairs of pixels or voxels with predefined gray level intensities in different directions.

(4) Grey Level Dependence Matrix (GLDM): quantifies gray level dependencies in an image.

(5) Grey Level Run Length Matrix (GLRLM): describes information about the spatial distribution of consecutive pixels having the same gray level value.

(6) Grey Level Size Zone Matrix (GLSZM): based on a similar principle to the GLRLM, but counts of the number of groups (so-called zones) of connected voxels that share the same gray level intensity.

The derived images were obtained by wavelet filtering for high pass or low pass filter (wavelet-LHL, wavelet-LHH, wavelet-HLL, wavelet-LLH, wavelet-HLH, wavelet-HHH, wavelet-HHL and wavelet-LLL) and Laplacians of Gaussians filtering with different sigma value (log-sigma-[1.0, 2.0, 3.0, 4.0 or 5.0]-mm-3D) from the original images. Detailed descriptions and mathematic formulas of each radiomics feature are shown on https://pyradiomics.readthedocs.io/en/latest/features.html.

Appendix E2: Detailed descriptions of selected radiomics features

Finally, 11 features were screened for developing radiomics models. The heatmap and correlation heatmap of these features are shown in Supplementary Figure 2 and Supplementary Figure 3. A brief description of those radiomic features is shown in following table.

Image type	Feature type	Feature name	Descriptions
original	glszm	LargeAreaLowGrayLevelEmphasis	measures the proportion in the image of the joint distribution of larger size zones with lower gray-level values.
log-sigma-1-0-mm-3D	glszm	SmallAreaEmphasis	a measure of the distribution of small size zones, with a greater value indicative of more smaller size zones and more fine textures.
log-sigma-2-0-mm-3D	glszm	SmallAreaEmphasis	a measure of the distribution of small size zones, with a greater value indicative of more smaller size zones and more fine textures.
log-sigma-4-0-mm-3D	glszm	SizeZoneNonUniformityNormalized	measures the variability of size zone volumes throughout the image, with a lower value indicating more homogeneity among zone size volumes in the image.
log-sigma-5-0-mm-3D	glszm	GrayLevelNonUniformity	measures the variability of gray-level intensity values in the image, with a lower value

			indicating more homogeneity in intensity values.
log-sigma-5-0-mm-3D	glcm	Idn (Inverse Difference Normalized)	quantifying the local homogeneity of an image
wavelet-LLH	firstorder	Median	the median gray level intensity within the ROI.
wavelet-LLH	glcm	Imc1 (Informational Measure of Correlation 1)	quantifying the complexity of the texture
wavelet-HLH	glcm	Imc2 (Informational Measure of Correlation 2)	also quantifying the complexity of the texture
wavelet-HLL	glszm	SmallAreaLowGrayLevelEmphasis	measures the proportion in the image of the joint distribution of smaller size zones with lower gray-level values.
wavelet-HLH	glszm	LowGrayLevelZoneEmphasis	measures the distribution of lower gray-level size zones, with a higher value indicating a greater proportion of lower gray-level values and size zones in the image.

Appendix E3: The hyperparameters of each machine learning model

1. LR

'C': 0.056, 'class_weight': {}, 'dual': False, 'fit_intercept': True, 'intercept_scaling': 1, 'l1_ratio': None, 'max_iter': 1000, 'multi_class': 'auto', 'n_jobs': None, 'penalty': 'l2', 'solver': 'lbfgs', 'tol': 0.0001, 'verbose': 0, 'warm_start': False

2. SVM

'C': 1.0, 'break_ties': False, 'cache_size': 200, 'class_weight': None, 'coef0': 0.0, 'decision_function_shape': 'ovr', 'degree': 3, 'gamma': 'auto', 'kernel': 'rbf', 'max_iter': -1, 'probability': True, 'shrinking': True, 'tol': 0.001, 'verbose': False

3. KNN

'algorithm': 'auto', 'leaf_size': 30, 'metric': 'minkowski', 'metric_params': None, 'n_jobs': -1, 'n_neighbors': 10, 'p': 2, 'weights': 'uniform'

4. XGBoost

'objective': 'binary:logistic', 'base_score': None, 'booster': 'gbtree', 'enable_categorical': False, 'eval_metric': None, 'learning_rate': None, 'max_bin': None, 'max_cat_threshold': None, 'max_depth': 2, 'max_leaves': None, 'min_child_weight': None, 'missing': nan, 'monotone_constraints': None, 'multi_strategy': None, 'n_estimators': 10, 'n_jobs': -1, 'num_parallel_tree': None, 'tree_method': 'auto', 'validate_parameters': None, 'verbosity': 0

5. RF

'max_depth': 4, 'n_estimators': 130, 'min_samples_split': 9, 'min_samples_leaf': 5, 'bootstrap': True, 'ccp_alpha': 0.0, 'class_weight': 'balanced_subsample', 'criterion': 'entropy', 'max_features': 'log2', 'max_leaf_nodes': None, 'max_samples': None, 'min_impurity_decrease': 0.0002, 'min_weight_fraction_leaf': 0.0, 'n_jobs': -1, 'oob_score': False, 'verbose': 0, 'warm_start': False

Supplementary Tables

Table S1 The CT protocols

CT version	Discovery CT750 HD	Revolution CT	Brilliance iCT	SOMATOM Definition Flash	SOMATOM Definition AS	Aquilion One
CT tube voltage	120 kV	120 kV	120 kV	120 kV	120 kV	120 kV
CT tube current	200-500 mA	100-250 mA	320 mA	250 mA	110-250 mA	250 mA
Gantry rotation time	0.60s	0.28s	0.27s	0.50s	0.50s	0.50s
Detector collimation (mm)	0.625 mm	0.625 mm	0.625 mm	0.6 mm	0.6 mm	0.5 mm
Image matrix	512*512	512*512	512*512	512*512	512*512	512*512
Slice thickness	5-7 mm	1-5 mm	5 mm	1-5 mm	1-5 mm	1-7 mm
Centers	Center 1	Center 2; Center 3	Center 3	Center 1	Center 2	Center 4

Table S2 Comparisons of AUC bewteen models by Delong's test.

Model	Training set		Test set	
	AUC	P value	AUC	P value
RF*	0.965	-	0.815	-
LR	0.829	<0.001	0.803	0.847
SVM	0.917	0.035	0.709	0.081
KNN	0.827	<0.001	0.679	0.045
XGBoost	0.961	0.643	0.794	0.602

* Delong's test was used for AUC comparison between RF with LR, SVM, KNN and XGBoost models, respectively.

Supplementary Figures

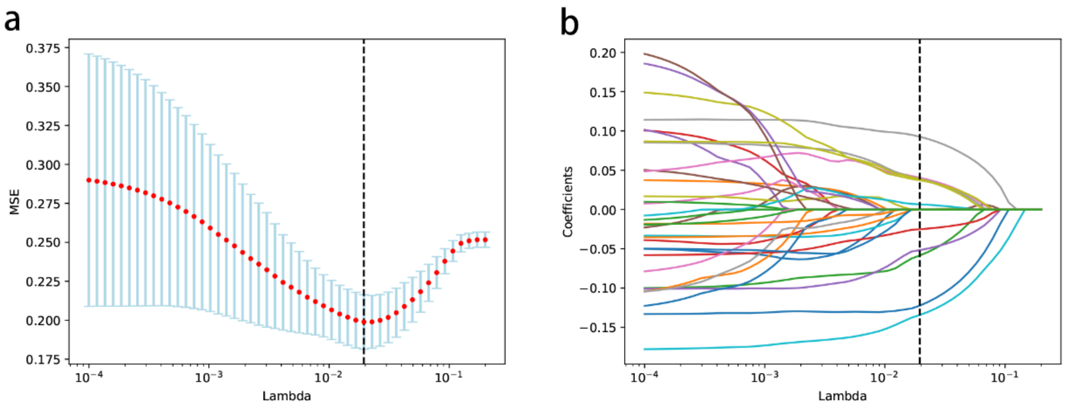

Fig. S1 The process of radiomics feature selection. (A) A total of 11 significant radiomics features were identified and selected by LASSO regression model in Tuning parameter (λ) of 0.0194. (B) A coefficient profile plot was generated versus the selected log λ value.

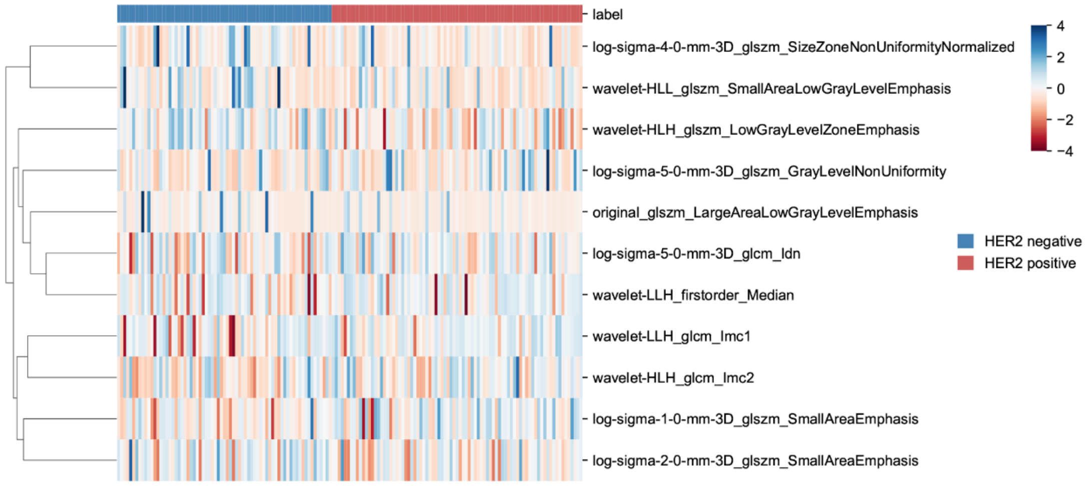

Fig. S2 The heatmap of 11 radiomics features selected by the LASSO regression algorithm for differentiation between HER2-positive and HER2-negative bladder cancer.

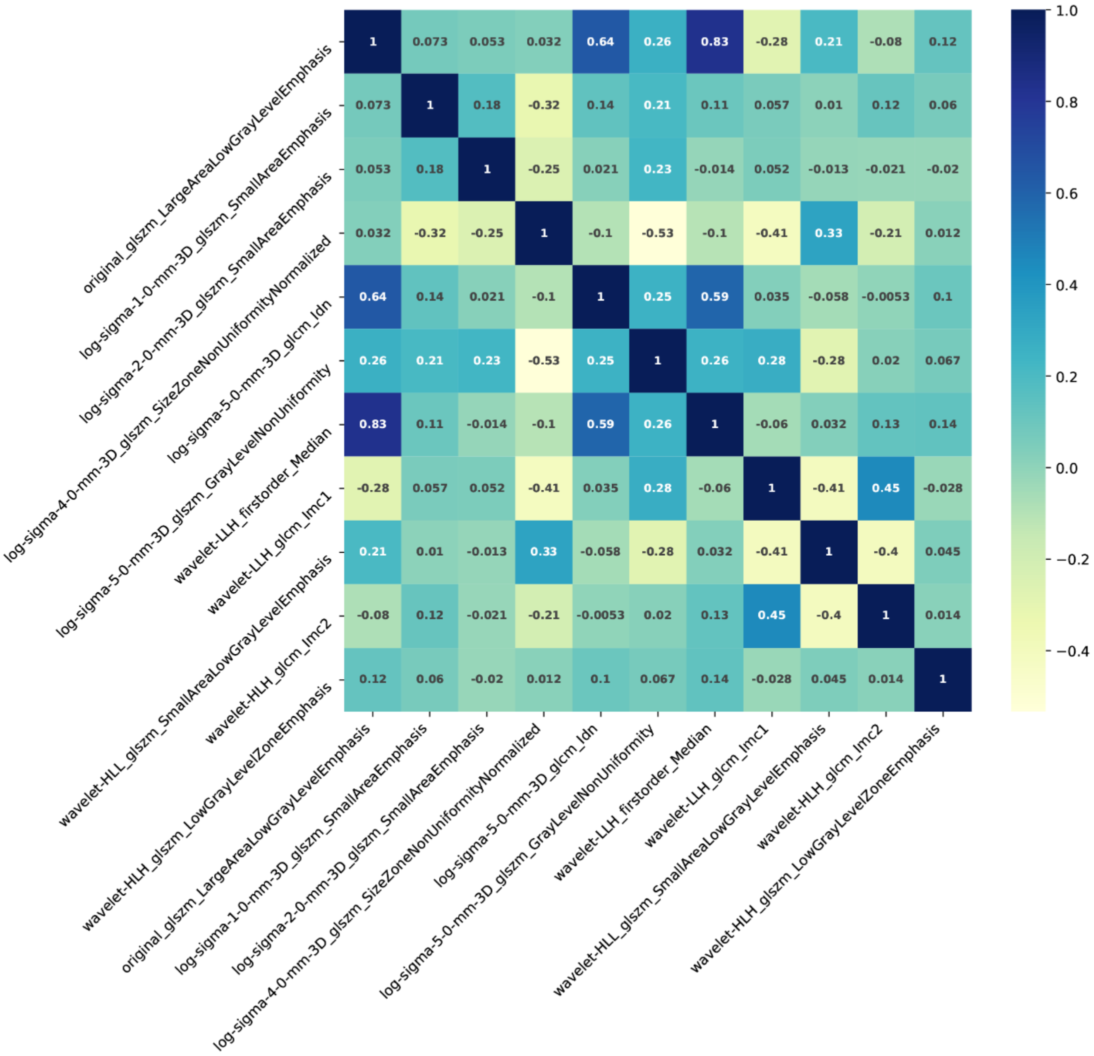

Fig. S3 Heatmap of correlation between radiomics features selected by the LASSO regression algorithm

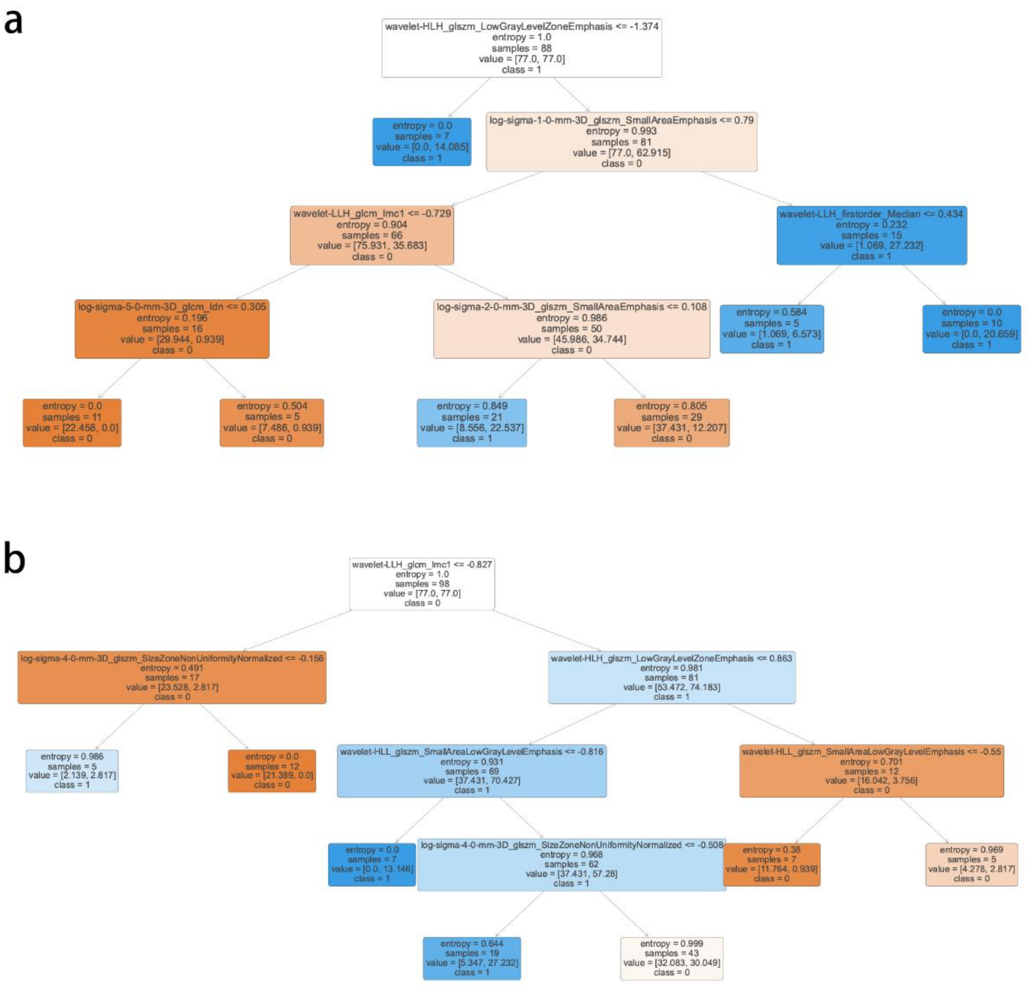

Fig. S4 Visualization of two decision trees in the Random Forest (RF) model. RF is an ensemble model that aggregates multiple binary decision trees. In a decision tree, each node represents a feature-based condition, which is selected to divide the dataset into two subsets, ensuring that similar samples are grouped together. This process facilitates the identification of predictive patterns by individual trees, thereby improving the overall accuracy and robustness of the model.
